# Animal protein intake is directly associated with serum level of pentraxin 3 in hemodialysis patients

**DOI:** 10.1038/s41598-023-48671-8

**Published:** 2023-12-07

**Authors:** Fatemeh Navab, Sahar Foshati, Mohammad Bagherniya, Gholamreza Askari, Firouzeh Moeinzadeh, Alieh Gholaminejad, Cain C. T. Clark, Mohammad Hossein Rouhani

**Affiliations:** 1 Nutrition and Food Security Research Center, Department of Community Nutrition, School of Nutrition and Food Science, Isfahan University of Medical Sciences, Isfahan, Iran; 2 Nutrition Research Center, Department of Clinical Nutrition, School of Nutrition and Food Sciences, Shiraz University of Medical Sciences, Shiraz, Iran; 3 Isfahan Kidney Diseases Research Center, Isfahan University of Medical Sciences, Isfahan, Iran; 4 Regenerative Medicine Research Center, Faculty of Medicine, Isfahan University of Medical Sciences, Isfahan, Iran; 5 Centre for Intelligent Healthcare, Coventry University, Coventry, CV1 5FB UK

**Keywords:** Nutrition, End-stage renal disease

## Abstract

Inflammation plays an important role in Cardiovascular disease (CVD) pathogenesis as the main cause of mortality in hemodialysis (HD) patients. Despite the relevance of nutrition and dietary intakes for inflammation status, the role of dietary protein sources remains unclear. The aim of this study was to evaluate the association between the different types of dietary protein and pentraxin 3 (PTX3) levels in HD patients. In this multi-center cross-sectional study, 227 adult patients undergoing HD for a minimum 90 days were recruited. A validated 168-item food frequency questionnaire was used to assess dietary intakes. Also, 5 ml blood samples were collected from each patient to measure the concentration of serum PTX3. Overall, 227 patients, including 63 women and 164 men, with a mean age of 58 years, participated in this study. There was a greater intake of animal protein per kilogram dry weight among patients with higher levels of PTX3 (0.46 vs. 0.54 g/kg; P = 0.035). In contrast, consumption of total protein and plant protein per kilogram dry weight was not different across PTX3 levels. Moreover, the chance of increased PTX3 concentration was directly associated with a one-unit increase in animal protein intake per kilogram dry weight, after adjusting for confounders. We did not observe any association between one-unit increases in plant protein intake per kilogram dry weight and chance of increased PTX3. In conclusion, animal protein intake was directly associated with circulating PTX3.

## Introduction

Hemodialysis (HD) is the main form of renal replacement therapy for most patients with end-stage renal disease (ESRD)^1^. Unfortunately, the mortality rate in HD patients is significantly higher than the general population^2^. Epidemiologic studies showed that cardiovascular diseases (CVDs) are the main cause of the mortality in HD patients^3^, whilst inflammation plays an important role in CVD pathogenesis among HD patients^4^, which can lead to adverse clinical outcomes^5,6^. Inflammatory markers are commonly used in early diagnosis, prognosis, and monitoring of renal disease and considered as a predictor of mortality^7^. Pentraxin 3 (PTX3) is a novel inflammatory biomarker that has been assessed in recent studies^8^. PTX3 as a member of the pentraxin superfamily, and responsible for modulating immune-inflammatory responses^9^. As opposed to nonspecific biomarkers of inflammation, including C-reactive protein (CRP) and serum amyloid P mainly produced by hepatic cells^10^, production of PTX3 occurs in cells which have a contribution to the onset and progression of CVD, such as vascular endothelial cells, fibroblasts, smooth muscle cells, epithelial cells, myeloid cells, and lymphatic cells^9^. Therefore, PTX3 is considered as a specific and sensitive indicator that can connect inflammation to CVD^11^. Moreover, PTX3 is related to renal functions^12,13^ and considered as an independent predictor of mortality among CKD patients^14^. Previous study reported that Spearman's rank correlation coefficient between PTX3 and CRP was 0.157 (P = 0.013) in patients with ESRD^15^.

Nutrition and dietary intakes are among the most important factors influencing the inflammation status in HD patients^16^. Malnutrition, as a cause of inflammation, is prevalent among these patients, resultant from decreasing amounts of dietary protein intake to slow down progression of chronic kidney disease (CKD)^17^. Nevertheless, the role of dietary protein sources in inflammation status has been neglected^18^. Results of a recent meta-analysis demonstrated a favorable relationship between a healthy dietary pattern (i.e., consuming more plant proteins and fish, along with less red meat) and mortality risk in CKD patients^19^. Moreover, a vegetarian diet can be recommended to adults with CKD because it can provide more protein with less bioavailable phosphorus^20^, and reduce uremic toxin levels^21^, hypertension^22^, metabolic acidosis^23^, and inflammation^24^. A systematic review and meta-analysis reported that in comparison with total protein intake, animal proteins elevated CRP concentration. Also, a decreasing trend in CRP was reported when plant proteins compared with animal proteins in adults with CKD^25^.Although previous studies have showed that animal protein had adverse effects on CRP concentration, the association between the sources of dietary protein and levels of PTX3 has not been assessed in HD patients. Therefore, we aimed to evaluate the association between different types of dietary protein (animal vs. plant) and levels of circulating PTX3.

## Materials and methods

### Sample size calculation

Sample size was estimated by following formula^26^: N = [(Z_1−α/2_)^2^ × SD^2^]/day^2^. PTX3 was used as the main variable to calculate the sample size. According to previous studies conducted in Iran on HD patients, the standard deviation of PTX3 was equal to 2.5 ng/ml^27^. Also, α was defined as 0.05, and d = 0.33 ng/ml. Therefore, minimum required sample size for this study was 221.

### Study design and population

From September 2021 to March 2022, we carried out a multi-center cross-sectional investigation on adult maintenance HD patients. Individuals were selected from five different HD centers in Isfahan, Iran. Those who met the following criteria were included: (1) being on maintenance HD for a minimum of 90 days; (2) aged 18 or older and any gender; and (3) being able and eager to take part in survey. In contrast, patients were excluded if they: (1) had enteral or parenteral nutrition; and (2) reported daily energy intakes lower than 800 kcal/day (3347 kJ) or more than 4200 kcal/day (17,572.8 kJ) (because of over-under reporting)^28^; (3) were smokers; and (4) recorded history of myocardial infarction. Informed consent was obtained from all participants, after a brief description of the study's significance and protocol, prior to study commencement. The study protocol was approved by The Research Ethics Committee of Isfahan University of Medical Sciences, Isfahan, Iran (IR.MUI.RESEARCH.REC.1399.605). All methods were performed in accordance with the relevant guidelines and regulations.

### Dietary assessment

The usual dietary intake during the past year was evaluated using a 168-item semi-quantitative food frequency questionnaire (FFQ). FFQs were filled out via in-person interviews by an experienced dietitian. This questionnaire contained a standard portion size for each food item along with nine options for frequency of consumption on a daily, weekly or monthly basis. Finally, analyzing dietary intakes was performed by converting reported food intakes to gram per day using household measures^29^. Then measuring macro- and micro-nutrients intake was performed by Nutritionist IV software (First Databank, Hearst Corp). Previously, the validity and reliability of this questionnaire were assessed and shown to be acceptable in the Iranian population^30^.

### Laboratory assessments

A venous blood sample (5 mL) was collected from each participant. Then, serum was separated by centrifuging (2000 rpm for 10 min at 4 °C). The concentration of serum PTX3 was measured by using enzyme-linked immunosorbent assay (ELISA) kits (ZellBio GmbH, Germany) based on the Biotin double antibody sandwich technology (Inter Assay PTX3: CV < 10%, Intra Assay PTX3: CV < 12%).

### Anthropometric measurements

To measure dry weight and height, we used a Seca scale and inelastic tape, respectively (Seca Co., Hamburg, Germany). Weight measurements were performed when patients had light clothes, measured to the nearest 0.1 kg. Also, height was assessed when subjects were in standard, upright position, and unshod, measured to the nearest 0.1 cm (cm). Dry weight was defined as the minimum acceptable weight following the dialysis session without exhibiting any signs or symptoms of hypovolemia or hypervolemia^31^. In order to calculate the body mass index (BMI), dry weight was divided by squared height. BMI < 23 kg/m^2^ was the criterion of the malnutrition^32^. Addition, mid-upper arm circumference (MUAC), waist circumference (WC), as well as hip circumference (HC), were measured by an inelastic tape, with 0.1 cm accuracy, with participants in a standing position. The MUAC measurement was performed at an equidistant point between the inferior border of the acromion process and the tip of the olecranon process on the bare left arm^33^. For WC, the tape was placed at the midpoint between the iliac crest and lowest rib^34^ and HC was obtained at the maximum circumference of the buttocks^35^. The waist-to-hip ratio (WHR) was calculated by dividing the WC by the HC.

### Assessment of Quality of HD:

To assess dialysis adequacy, Kt/V and Urea reduction ratio (URR) values were used. URR was calculated using the following equation^36^:

$${\text{URR}} = \left[ {{{\left( {{\text{Blood urea nitroge}}{{\text{n}}_{{\text{predialysis}}}} - {\text{Blood urea nitroge}}{{\text{n}}_{{\text{postdialysis}}}}} \right)} \mathord{\left/ {\vphantom {{\left( {{\text{Blood urea nitroge}}{{\text{n}}_{{\text{predialysis}}}} - {\text{Blood urea nitroge}}{{\text{n}}_{{\text{postdialysis}}}}} \right)} {{\text{Blood urea nitroge}}{{\text{n}}_{{\text{predialysis}}}}}}} \right. \kern-\nulldelimiterspace} {{\text{Blood urea nitroge}}{{\text{n}}_{{\text{predialysis}}}}}}} \right] \times 100$$

The second quality measure of HD was Kt/V (where K represents the urea clearance amount, t represents the dialysis time, and V represents the urea distribution volume)^36^.

### Assessment of other variables

Patients' general characteristics, such as age, job, and marital status, were obtained through verbatim questions. In addition, the medical information, including causes of renal disease, comorbidities, dialysis duration, the number of dialysis sessions per week, dialysis vintage, as well as medications, were collected from hospital records.

### Statistical analysis

The normal distribution of data was evaluated by Q–Q plot, histogram chart, Skewness statistic, and Kolmogorov–Smirnov test. Numerical variables were presented as mean ± standard deviation (SD), whereas categorical data were shown as numbers (percentages). Analyzing categorical and continuous variables across PTX3 levels was carried out using Chi-square tests and one-way analysis of variance (ANOVA), respectively. The association between PTX3 and intake of different types of dietary protein in HD patients was assessed using logistic regression. A wide range of cutoffs for PTX3 are reported for preventing different diseases. For example, in sever sepsis and fatal disease in bacteremic PTX3 cutoffs are 14.1 ng/ml and 15 ng/ml, respectively^37,38^. In HD patients, the best reported cutoff for PTX3 to predict morbidity and mortality were 0.55 ng/ml and 0.25 ng/ml, respectively^39^. In our study, the lowest concentration of PTX3 was 1.15 ng/ml. Therefore, we could not use this cutoff. As there is not an approved cut-off point for PTX3 in HD patients, we use the median-cut method. The odds ratios (ORs), with 95% confidence interval (CI), was reported for different adjusted models. In the first model, general confounders including age and sex were adjusted. As the independent variable (dietary protein intake) was strongly depends on energy intake, we also included calorie intake as a covariate in Model 1. In Model 2, we included remained variables based on the literature review including dialysis frequency, dialysis duration, urea reduction ratio, waist circumference, hip circumference, arm circumference, height, and cause of renal disease. We used SPSS version 21 for all statistical analyses. P < 0.05 was considered to be statistically significant.

### Ethics approval and consent to participate

This study was ethically approved by The Research Council and Ethical Committee of Isfahan University of Medical Sciences, Isfahan, Iran, (Code: IR.MUI.RESEARCH.REC.1399.605). Also, all participants completed an informed consent form. Mohammed Hossein Rouhani as the lead author affirms that this manuscript is an honest, accurate, and transparent account of the study being reported; that no important aspects of the study have been omitted; and that any discrepancies from the study as planned have been explained.

## Results

Overall, 227 patients, including 63 women and 164 men, with the mean age of 58 years old, were included in the present study. As shown in Table 1, the general characteristics of participants were categorized according to PTX3 levels. There were no significant differences in sex (P = 0.860), age (P = 0.952), marital status (P = 0.728), employment status (P = 0.275), dry weight (P = 0.698), height (P = 0.372), BMI (P = 0.915), malnutrition (P = 0.667), waist circumference (P = 0.242), hip circumference (P = 0.376), waist to hip ratio (P = 0.379), arm circumference (P = 0.516), dialysis vintage (P = 0.910), dialysis frequency (P = 0.114), dialysis duration/session (P = 0.193), Kt/V (P = 0.568), URR (P = 0.378), cause of renal failure (P = 0.398) between low levels (< 3.75 ng/mL), and high levels (≥ 3.75 ng/mL) of PTX3.

**Table 1:** General characteristics of hemodialysis patients across levels of pentraxin 3.

Variables	Pentraxin 3 > Median (< 3.75 ng/mL)	Pentraxin 3 ≥ Median (≥ 3.75 ng/mL)	P value
N	112	115	
Demographic variables			
Sex (% male)	73.2	72.2	0.860
Age (years)	58.86 ± 15.46	58.00 ± 16.05	0.952
Marital status (% married)	85.3	83.5	0.728
Employment status (%)			
Self-employed	6.3	13.9	0.275
Retired	34.8	33	
Un-employed	31.3	26.1	
Others	27.7	27	
Dry weight (kg)	66.06 ± 14.76	66.89 ± 14.40	0.698
Height (cm)	164.29 ± 9.24	165.50 ± 9.88	0.372
Body mass index (kg/m^2^)	24.40 ± 4.80	24.48 ± 4.62	0.915
Malnutrition^1^ (%)	40	43	0.667
Waist-circumference (cm)	93.53 ± 13.74	95.69 ± 13.66	0.242
Hip-circumference (cm)	96.69 ± 10.35	97.90 ± 9.91	0.376
Waist to hip ratio	0.96 ± 0.08	0.97 ± 0.09	0.379
Arm-circumference (cm)	27.96 ± 3.96	28.30 ± 3.92	0.516
Dialysis vintage (W)	48.68 ± 46.06	49.33 ± 40.36	0.910
Dialysis frequency (%)			
1 × per week	9.8	2.6	0.114
2 × per week	11.6	15.7	
3 × per week	76.8	80.9	
4 × per week	1.8	0.9	
Dialysis duration/session (h)	4.017 ± 0.88	3.90 ± 0.28	0.193
Kt/V	1.31 ± 0.23	1.32 ± 0.24	0.568
URR	0.71 ± 0.15	0.73 ± 0.17	0.378
Cause of renal failure (%)			
Diabetes mellitus	31.3	21.7	0.398
Hypertension	28.6	29.6	
Acute kidney injury	1.8	2.6	
Nephrolithiasis	0.9	0.9	
Multi causes	14.2	20	
Others	23.2	25.2	

Data are presented as mean ± SD for continuous and percent for categorical variables.

P-value obtained from chi-square analysis for categorical variables and Independent t-test for continuous variables.

*URR* Urea reduction ratio.

^1^Body mass index < 23 kg/m^2^^32^.

Energy-adjusted nutrient intakes across different PTX3 concentrations are shown in Table 2. No significant differences were observed in intake of carbohydrate (P = 0.493), protein (P = 0.073), fat (P = 0.967), total fiber (P = 0.719), vitamin A (P = 0.006), vitamin C (P = 0.152), vitamin E (P = 0.421), and vitamin D (P = 0.179), calcium (P = 0.247), thiamin (P = 0.547, riboflavin (P = 0.090), niacin (P = 0.386), and phosphorus (P = 0.330) across different levels of PTX3.

**Table 2:** Dietary intake of hemodialysis patients across levels of pentraxin 3.

Nutrients	Pentraxin 3 < Median (< 3.75 ng/mL)	Pentraxin 3 ≥ Median (≥ 3.75 ng/mL)	P
Energy (Kcal/day)	1824.31 ± 856.64	1797.70 ± 879.25	0.818
Carbohydrate (g/day)	274.43 ± 116.91	266.52 ± 129.38	0.493
Protein (g/day)	65.33 ± 28.46	68.82 ± 41.35	0.073
Fat (g/day)	58.24 ± 51.61	57.01 ± 31.99	0.967
Total fiber (g/day)	32.67 ± 16.13	32.84 ± 17.56	0.719
Vitamin A (RAE)	463.38 ± 286.81	550.31 ± 418.12	0.006
Vitamin C (mg)	135.66 ± 107.66	147.53 ± 110.21	0.152
Vitamin E (mg)	11.51 ± 14.54	10.39 ± 5.13	0.421
Vitamin D (µg)	0.72 ± 0.60	0.82 ± 0.95	0.179
Calcium (mg)	1096.34 ± 608.73	1155.95 ± 771.78	0.247
Thiamin (mg)	1.46 ± 0.63	1.41 ± 0.73	0.547
Riboflavin (mg)	1.58 ± 0.72	1.68 ± 1.02	0.090
Niacin (mg)	18.03 ± 8.19	18.47 ± 11.04	0.386
Phosphorus (mg)	1169.82 ± 497.53	1192.55 ± 673.03	0.330

Data are presented as mean ± SD.

P-value obtained from analysis of covariance (ANCOVA) adjusted for total calorie intake.

Mean intake of different types of dietary protein per kilogram dry weight across PTX3 levels is presented in Table 3. Patients with higher levels of PTX3 consumed significantly more animal protein per kilogram dry weight (0.46 vs. 0.54 g/kg; P = 0.035). Intake of total protein and plant protein per kilogram dry weight was not different across levels of PTX3.

**Table 3:** Mean intake of different types of dietary protein per kilogram dry weight across levels of pentraxin 3 in hemodialysis patients.

Dietary protein	Pentraxin 3 > Median (< 3.75 ng/mL)	Pentraxin 3 ≥ Median (≥ 3.75 ng/mL)	P
Total protein (kg)	1.00 ± 0.3	1.07 ± 0.3	0.122
Animal protein (kg)	0.46 ± 0.2	0.54 ± 0.2	0.035
Plant protein (kg)	0.54 ± 0.2	0.53 ± 0.2	0.775

Data are presented as mean ± SD.

P-value obtained from analysis of covariance (ANOVA) adjusted for total calorie intake.

Results of the association between chance of elevated PTX3 and one-unit increase in intake of different types of dietary protein per kilogram dry weight are reported in Table 4. Although a one-unit increase in intake of total protein intake per kilogram dry weight was not associated with the chance of increased PTX3 in crude model and Model 1, it was marginally, significantly related to the chance of elevated PTX3 levels in Model 2 (OR = 3.114; 95% CI 0.943, 10.283; P = 0.062). Also, we observed a direct association between a one-unit increase in intake of animal protein intake per kilogram dry weight and chance of increased PTX3 concentration in the Model 1 (OR = 3.123; 95% CI 1.077, 9.053; P = 0 0.036) and Model 2 (OR = 4.524; 95% CI 1.247, 16.417; P = 0.022). A one-unit increase in intake of plant protein intake per kilogram dry weight were not related to the chance of increased PTX3, before or after adjusting for potential confounders.

**Table 4:** The association between odds ratio for elevated pentraxin 3 (> Median) and one-unit increase in intake of different types of dietary protein per kilogram dry weight in hemodialysis patients.

	Models	Odds ratio	95% CI		P
			Lower	Upper	
Total protein (kg)	Crude	1.193	0.746	1.909	0.462
	Model 1	1.934	0.839	4.461	0.122
	Model 2	3.114	0.943	10.283	0.062
Animal protein (kg)	Crude	1.778	0.820	3.857	0.145
	Model 1	3.123	1.077	9.053	0.036
	Model 2	4.524	1.247	16.417	0.022
Plant protein (kg)	Crude	0.849	0.353	2.043	0.715
	Model 1	0.823	0.219	3.100	0.774
	Model 2	0.752	0.0126	4.478	0.754

P-value obtained from logistic regression.

Model 1 was adjusted for age, sex, total calorie intake.

Model 2: was adjusted for age, sex, total calorie intake, dialysis frequency, dialysis duration, urea reduction ratio, waist Circumference, hip Circumference, arm Circumference, height, cause of renal disease.

## Discussion

In accord with the aim of this study, we found that subjects with higher levels of PTX3 consumed significantly more animal protein per kilogram dry weight. Also, a one-unit increase in intake of animal protein per kilogram dry weight was directly associated with chance of increased PTX3.

Permanent low-grade inflammation is a concern in ESRD^40^, which can lead to exacerbated mortality and morbidity risk^41^. The inflammation experienced by CKD patients is traditionally monitored via interleukin-6, tumor necrosis factor alpha, and CRP^42–44^. Compared to these inflammatory biomarkers, PTX3 is considered to be a better predictor of inflammation due to its expression in wide range of tissues^45^. Also, PTX3 concentration is positively related to conventional inflammatory biomarkers^45^. In contrast to CRP, release of PTX3 occurs quickly from neutrophil granules in response to inflammatory signals^46^. The sensitivity of PTX3 to a micro inflammatory process is greater than other inflammatory biomarkers in HD patients^47^. Thus, PTX3 is accepted as a quick and sensitive indicator of dialysis-related inflammation among HD patients^47^.

Despite the importance of dietary protein intake in HD patients, the Kidney Disease Outcomes Quality Initiative (KDOQI) did not declare any recommendation regarding the type of protein (plant versus animal) due to the insufficiently powered studies^48^. Nephrologists typically avoid vegetarian-based diets^49^, as it has been traditionally believed that these kinds of diets are not only nutritionally inadequate, but also dangerous due to their high potassium contents^50^. Nevertheless, there is strong evidence advocating that plant based diets can be nutritionally sufficient and beneficial if they are well-balanced and varied^51–53^. On the other hand, no studies have demonstrated significant differences in serum potassium levels in a comparison of potassium intakes from plant based diets versus omnivorous diets^50^. Furthermore, some studies have shown the beneficial effects of plant protein or plant-based diets, including Dietary Approaches to Stop Hypertension (DASH), Mediterranean, or vegetarian diets, in reduction of inflammatory markers, as well as improvement of complications related to various chronic diseases^54,55^. Plant-based diets are rich in antioxidants and vitamins, whereas animal protein-rich diets, particularly red and processed meat, generally contain high levels of sodium and saturated fatty acids, which can negatively impact on kidney functions^56^. Results of a recent meta-analysis on CKD patients revealed a positive association between consumption of animal protein and CRP concentration^25^. In contrast, results regarding other biomarkers of inflammation are controversial. Indeed, a previous investigation reported that animal protein may reduce the concentration of pro-inflammatory adipokines including chemerin and progranulin^57^. Therefore, it is necessary to perform a comprehensive study regarding the association between type of dietary protein and biomarkers of inflammation to elucidate a pattern that shows an overall conclusion. It would also be prudent to determine which inflammatory biomarkers are the most reliable predictor(s) of adverse outcomes in HD patients.

There are several posited mechanisms regarding the association between inflammation and animal/plant protein intake. First, in comparison to plant protein, animal protein intake can negatively impact on the gut microbiome composition by producing greater amount of ammonia and sulfur-based materials. As a result of gut microbiome imbalance, promotion of inflammation will occur^58–61^. While a plant-based diet increases the production of short-chain fatty acids, which have beneficial effects on improvement of the dysbiotic microflora compositions, they can result in the inhibition of the pathogens growth, reduction of pro-inflammatory parameters, decreased oxidative stress, and lower uremic toxins^62,63^. Second, the cholesterol derived from animal fat and meat plays an important role in the development of inflammation^64–66^. PTX3 expression can induce both locally and systemically by circulating levels of LDL cholesterol (a pivotal mediator of atherosclerosis)^67^. Third, sulfur-containing amino acids found in animal proteins can enhance dietary acidity and result in exacerbating metabolic acidosis in CKD patients^68^. Dietary acid load can induce inflammation^69^. Meanwhile, metabolization of plant proteins is associated with a greater consumption of hydrogen ions^70^ and greater production of bicarbonate in order to minimize acid productions^71^. Consequently, production of inflammatory markers, oxidative stress, and uremic toxins may be reduced through this process^72,73^.

We have presented a novel addition to the literature, highlighting that the type of protein consumed should be acutely considered in HD patients. Nevertheless, addressing the limitations associated with the present study is important. First, despite controlling for multiple confounders, residual confounders may remain, and can only be elucidated in further work. Second, the cross-sectional design of this study precludes causal inferences being made. Concomitant to the limitations, there are several strengths that should be noted. Indeed, the use of an appropriate biomarker, consideration of potential confounders, the relatively large sample size, and the multicenter design of the study are inherent strengths of the present study. Reporting the results on a per kilogram dry weight basis is another advantage of this study. As all HD guidelines recommend a certain amount of protein per kilogram, our findings can be comparable with guidelines.

## Practical application

In conclusion, our findings suggest that, unlike consumption of plant protein, animal protein intake was significantly associated with increased circulating PTX3 levels among HD patients. Accordingly, extra consideration should be given the source(s) of protein intake in HD patients under clinical supervision.

### Supplementary Information

Supplementary Information.

## Data Availability

Data will be available on request. Please contact to following email: sm_rouhani@nutr.mui.ac.ir.
